# Supplementary material for: Resolving the full spectrum of human genome variation using Linked-Reads
Source: Genome Res. 2019 Apr;29(4):635–45. doi: 10.1101/gr.234443.118 (PMC6442396; doi:10.1101/gr.234443.118)
Supplement: Supplemental Material [file supp_gr.234443.118_Supplemental_longranger-2.2.zip › longranger-2.2.tar.bz2!/lr2.2-public/lib/stan/cmdstan/stan/doc/api/html/append__col_8hpp.html]

Stan: src/stan/math/matrix/append\_col.hpp File Reference


|  |  |
| --- | --- |
|  | Stan  2.5.0  probability, sampling & optimization |


- Main Page
- Related Pages
- Namespaces
- Classes
- Files

- File List
- File Members

All Classes Namespaces Files Functions Variables Typedefs Enumerations Enumerator Friends Macros Pages

- src
- stan
- math
- matrix

Namespaces |
Functions

append\_col.hpp File Reference

`#include <stan/math/matrix/Eigen.hpp>`  
`#include <stan/meta/traits.hpp>`  
`#include <stan/math/error_handling/matrix/check_size_match.hpp>`  
`#include <vector>`

Go to the source code of this file.

|  |  |
| --- | --- |
| Namespaces | |
|  | stan |
|  | Probability, optimization and sampling library. |
|  | |
|  | stan::math |
|  | Matrices and templated mathematical functions. |
|  | |

|  |  |
| --- | --- |
| Functions | |
| template<typename T1 , typename T2 , int R1, int C1, int R2, int C2> | |
| Matrix< typename return\_type  < T1, T2 >::type, Dynamic,   Dynamic > | stan::math::append\_col (const Matrix< T1, R1, C1 > &A, const Matrix< T2, R2, C2 > &B) |
|  | |
| template<typename T1 , typename T2 , int C1, int C2> | |
| Matrix< typename return\_type  < T1, T2 >::type, 1, Dynamic > | stan::math::append\_col (const Matrix< T1, 1, C1 > &A, const Matrix< T2, 1, C2 > &B) |
|  | |
| template<typename T , int R1, int C1, int R2, int C2> | |
| Matrix< T, Dynamic, Dynamic > | stan::math::append\_col (const Matrix< T, R1, C1 > &A, const Matrix< T, R2, C2 > &B) |
|  | |
| template<typename T , int C1, int C2> | |
| Matrix< T, 1, Dynamic > | stan::math::append\_col (const Matrix< T, 1, C1 > &A, const Matrix< T, 1, C2 > &B) |
|  | |


---

[ Stan Home Page ]

*© 2011–2014,
Stan Development Team.*
